# Correction to “Effects of Autophagy Inhibition by SAR405, a Selective VPS34 Inhibitor, on Pleural Mesothelioma Cells”

**DOI:** 10.1111/1759-7714.70336

**Published:** 2026-07-06

Y. Kuwabara , K. Sakai , K. Shiraishi , et al., “Effects of Autophagy Inhibition by SAR405, a Selective VPS34 Inhibitor, on Pleural Mesothelioma Cells,” Thoracic Cancer 17, no. 5 (2026): e70255, 10.1111/1759-7714.70255.41771171 PMC12952911

In the originally published version of this article, several errors were identified:

In Supporting Information Figure S1, the GAPDH (loading control) images for the H28, H2452, and 211H cell lines were inadvertently duplicated from Figure 1C of the main manuscript. Since Supporting Information Figure S1 represents independent experiments evaluating cisplatin treatment, the incorrect panels have been replaced with the correct images. The updates are as follows:

- **H28 cell line:** Both the p62 and GAPDH panels have been replaced with the correct images from the independent experiment performed on August 29, 2024.
- **H2452 cell line:** Both the p62 and GAPDH panels have been replaced with the correct images from the independent experiment performed on August 31, 2024.
- **211H cell line:** The GAPDH panel has been replaced with the correct corresponding loading control image from the experiment performed on October 16, 2024. The p62 panel is correct as originally published and was derived from an independent experiment performed on October 23, 2024; therefore, no change to the p62 panel is required.

In Section 3.4 (Results), the text incorrectly states: “*After 24h of treatment with 10.0 μM SAR405, Hoechst 33342 staining of H2452 cells revealed apoptotic morphology…*” This exposure duration should be corrected from “24h” to “48h” to maintain perfect consistency with Section 2.7 of the Methods and the Figure 6A legend, both of which correctly state that the Hoechst 33342 staining was performed after 48 hours of treatment.

In addition, clarification has been provided regarding Figures 1C and 6B, which were generated using aliquots from the same cell lysates obtained in the same experiments. Specifically, the western blots showing p62 expression in mesothelioma cell lines (H28, H2452, and 211H) treated with DMSO or 2.5, 5.0, or 10.0 μM SAR405 in Figure 1C, and the western blots showing the expression of apoptosis‐related proteins under the same treatment conditions in Figure 6B, were performed using aliquots of the same cell lysates. To further improve transparency, the following sentence should be added to the legend of Figure 6B, immediately after the existing legend text:

“Samples from the same cell lysates shown in Figure 1C were analyzed by western blotting.”

The authors apologize for any inconvenience caused.

